# Challenges in the Diagnosis and Treatment of Neuromyelitis Optica Spectrum Disorders: A Case Report With a Brief Review of Literature

**DOI:** 10.1177/2324709618809509

**Published:** 2018-11-01

**Authors:** Maria Elisa Morelli, Arianna Sartori, Antonio Bosco, Paolo Manganotti

**Affiliations:** 1 University Hospital and Health Services of Trieste, Trieste, Italy; 2 University of Trieste, Trieste, Italy

**Keywords:** neuromyelitis optica spectrum disorders, aquaporin-4 immunoglobulin G, short myelitis, rituximab

## Abstract

*Introduction*. According to the 2015 diagnostic criteria for neuromyelitis optica spectrum disorders (NMOSD), in aquaporin-4 immunoglobulin G (AQP4-IgG) seronegative patients, NMOSD can be diagnosed if stringent clinical and magnetic resonance imaging (MRI) criteria are fulfilled; however, in these cases, diagnostic and therapeutic challenges could arise. *Case Description*. A young man presented a severe, bilateral optic neuritis and paraparesis in an acute phase. MRI evidenced 3 spinal cord T1-contrast-enhanced (T1-Gd+) myelitic lesions, extending <3 vertebral segments. AQP4-IgG and oligoclonal bands were negative. Following a relapse, MRI showed 2 T1-Gd+ spinal cord lesions, extending <3 vertebral segments, and a T1-Gd+ lesion in both optic nerves, near their confluence in the chiasm. After administering rituximab, there were no new relapses, with the consequent improvement of the clinical and MRI lesions. *Conclusion*. The concurrent display of bilateral optic neuritis and short myelitis constitutes a “borderline” case. Rituximab may represent the most appropriate therapeutic choice possible cases of NMOSD.

## Introduction

Neuromyelitis optica spectrum disorders (NMOSDs) are autoimmune inflammatory diseases of the central nervous system (CNS). The prevalence of these diseases ranges from 2.6 to 3.6 cases per 10^5^ in Asians and from 0.7 to 4 cases per 10^5^ in Caucasians, and the median age of onset is 39 years.^1^ The most typical clinical features of NMOSD are optic neuritis (ON) and acute transverse myelitis. Less common clinical features could be area postrema syndromes, encephalopathy, and so on.^1^ A second relapse occurs within 1 year in 60% of patients and within 3 years in 90% of the cases, leading to severe disability.^1^ Serum aquaporin-4 immunoglobulin G (AQP4-IgG) is a reliable and potentially pathogenic biomarker of NMOSD (73% sensitivity, by cell-based assays, and 91% specificity). However, 10% to 25% of NMOSD patients are seronegative for AQP4-IgG (AQP4-IgG^neg^).^1^ The 2015 diagnostic criteria^2^ enable the detection of NMOSD in seropositive AQP4-IgG (AQP4-IgG^pos^) patients through the analysis of almost any CNS region. For AQP4-IgG^neg^ patients, detailed clinical, neuroimaging, and laboratory analyses are required to increase diagnostic accuracy, because these patients could be affected by multiple sclerosis (MS)/overlapping syndromes (eg, acute disseminated encephalomyelitis, neuro-systemic lupus erythematosus, Sjögren syndrome, neuro-Behçet disease, neurosarcoidosis, paraneoplastic and autoimmune encephalitis). An accurate diagnosis of such disorders has important treatment implications, because some MS immunotherapies could worsen NMOSD.^2^ Immunosuppressive agents are the best treatment strategy when there is suspicion of NMOSD, and rituximab, among others, is increasingly recognized as an established therapy for NMOSD, with long-term efficacy and an acceptable safety profile.^3,4^ The purpose of this case report is to show how difficult it can be to diagnose NMOSD based on stringent diagnostic criteria, and how it can be necessary to make a diagnosis after a careful differential diagnosis based on the typical clinical-radiological presentation of the disorder, in order to administer the most effective treatment possible with the lower risk of side effects.

## Case Presentation

**Figure 1.**
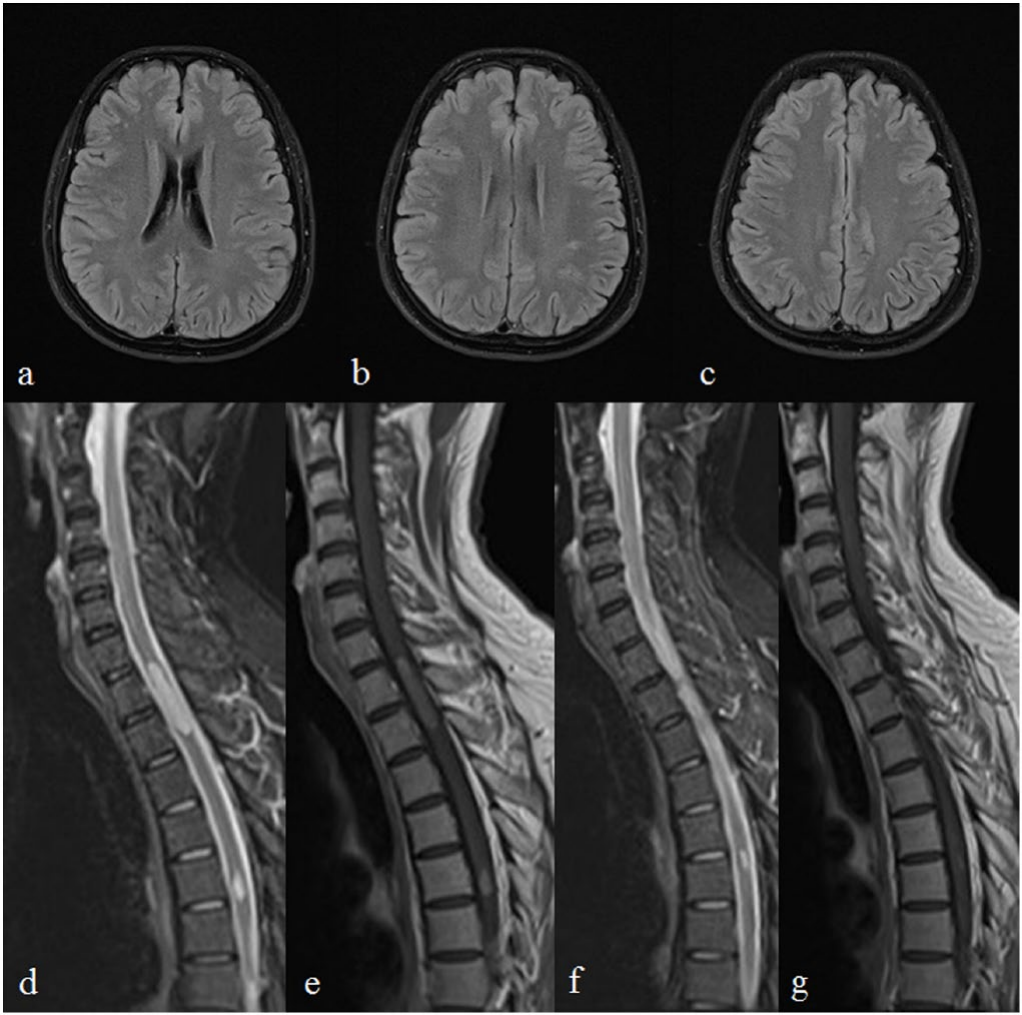
Brain and total spinal cord (SC) magnetic resonance imaging (MRI) performed at the onset of the disease, showing the following: a T2-hyperintense lesion (8 mm) in the left parietal subcortical white matter (WM), and other small, nonspecific, T2-hyperintense lesions in the subcortical WM, all of them without T1 contrast enhancement (a-c); 3 thoracic SC T2-hyperintense lesions, with extension <3 vertebral segments (VSs; respectively, located posteriorly to the T1-T2, T5, and T6 VSs) and T1-contrast-enhancement (d-g).

**Figure 2.**
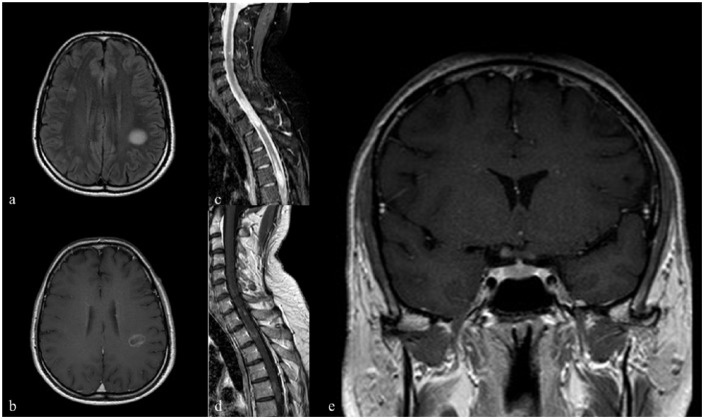
Brain and total spinal cord (SC) magnetic resonance imaging (MRI) performed at the relapse of the disease, showing the following: significant enlargement (23 mm) of the previous left parietal subcortical white matter (WM) lesion (a), with T1 ring contrast enhancement (b) (T1 scans not shown); T1 ring contrast enhancement of the thoracic SC lesion located posteriorly to the T1-T2 vertebral segments (VSs), confluence of the thoracic SC lesions located posteriorly to the T5 and T6 VSs in a single lesion located posteriorly to the T4-T6 VSs, with increase in extension (35 mm, <3 VSs) (c), and T1 ring contrast enhancement (d); a T1 contrast-enhanced lesion in both optic nerves, near their confluence in the chiasm, mostly on the right side (e).

We report a case of a 35-year-old man of Asian Indian ethnicity, born in Bangladesh and resident in Italy since 2001. No relevant diseases were detected in his past medical history. He had a family history of type 1 diabetes mellitus (father). He had worked as a painter for over 10 years, with possible exposure to toxic substances. However, he reported to have used protective air masks. On day 1, he reported frontal and binocular pain, and he had a drastic decline in visual acuity. He was hospitalized in the Ophthalmology Unit, where brain computed tomography scan, retinal fluorescein angiography, and standard bloodwork (kidney and liver function, fasting glucose, blood count, inflammatory markers, serum proteins, iron, vitamin B_12_, folate, and urinalysis) were carried out with normal results. Blood lead and trichloroethanol, as well as urinary methanol, trichloroacetic acid, and lead were undetectable. Signs of an existing systemic infectious disease were absent (Table 1). The analysis of visual-evoked potentials showed P100 waves of bilateral higher latency and amplitude at lower limit. A diagnosis of bilateral ON was performed, and a high-dose steroid treatment (intravenous methylprednisolone 1 g per day for 5 consecutive days) was started on day 3, with no improvement of visual acuity. Two days after the administration of high-dose steroids (day 5), the patient developed a rapidly worsening paraparesis, associated with bladder dysfunction. A brain and total (cervical, thoracic, lumbar, sacral segments) spinal cord (SC) magnetic resonance imaging (MRI) was performed. It showed the following: a T2-hyperintense, T1-isointense, T1-non-constrast-enhanced (T1-Gd−), and nonedematous lesion (8 mm) in the left parietal subcortical white matter (WM); other small, nonspecific, T2-hyperintense, and T1-Gd− lesions in the subcortical WM; 3 SC T2-hyperintense, T1-isointense, and T1-constrast-enhanced (T1-Gd+) lesions, with an oval morphology, an axial central position, and extension <3 vertebral segments (VSs; located in the thoracic SC, posteriorly to the T1-T2, T5, and T6 VSs; Figure 1a-g). On day 20, the patient was transferred to the Neurology Unit. The neurological examination showed that the gait was paraparetic (worse at the right lower limb) and was possible for only a few steps with monolateral assistance; the strength against maximum effort was reduced in all movements made with the lower limbs, more on the right than on the left side; a mild spasticity was present in the right lower limb; osteo-tendon reflexes were evocable with greater excitability in the right lower limb (with an evocable clonus at right ankle); a Babinski’s sign was present bilaterally; visual acuity was reduced to hand motion in both eyes; there were no deficits of orientation, cognition, motor functions, and osteo-tendon reflexes in the upper limbs, as well as in sensory, cerebellar, and other cranial nerve functions. Cerebrospinal fluid analysis showed a moderate increase of protein level (71 mg/dL), a normal cell count (4 cell/mm^3^), and the absence of oligoclonal bands (OBs). Signs of existing infectious disease of the CNS were absent (Table 1). Other autoimmune diseases (eg, acute disseminated encephalomyelitis, systemic lupus erythematosus, Sjögren syndrome, neuro-Behçet disease, sarcoidosis, primary angiitis of the CNS, paraneoplastic and autoimmune encephalitis) were excluded because of the absence of typical clinical, humoral, and radiological (MRI, chest X-ray) findings (Table 2). Serum neoplastic markers were in their normal ranges. Both serum myelin oligodendrocyte glycoprotein-IgG and AQP4-IgG (tested by cell-based assay) were negative; this result was confirmed also in a second analysis repeated 1 month later in a different laboratory. A second high-dose steroid treatment was started on day 20, with partial improvement of the paraparesis, but no effect on the visual deficit. On day 31, the patient’s visual acuity, bladder function, and paraparesis worsened, the patient could no longer ambulate, and there were also signs of bilateral hypesthesia, with a T4 sensory level, worse on the left side. A treatment with 1 cycle of therapeutic plasma exchange was started, with partial improvement of only visual and bladder symptoms. A new MRI of the brain, orbits, and total SC showed the following: a significant enlargement (23 mm) with ring contrast enhancement and T1-hypointensity of the previous left parietal subcortical WM lesion (Figure 2a and b); ring contrast enhancement of the thoracic SC lesion located posteriorly to the T1-T2 VSs, and confluence of the thoracic SC lesions located posteriorly to the T5 and T6 VSs in a single thoracic SC lesion located posteriorly to the T4-T6 VSs (<3 VSs), with increase in extension, T1-isointensity, and T1 ring contrast enhancement (Figure 2c and d); a tumefactive T2-hyperintense and T1-Gd+ lesion in both posterior optic nerves, near their confluence in the chiasm, mostly on the right side (Figure 2e). A third steroid cycle was started on day 39, followed by a significant improvement of the paraparesis, and a partial but promising effect on sensitive, visual, and bladder functions. In the days that followed, the patient started neurorehabilitation, and his gait progressively improved with bilateral assistance. Subsequently, a prophylactic treatment with rituximab was chosen and started with 1 g infusion on day 68 (month 2), followed by a second 1 g infusion 2 weeks later (day 82, month 3), with subsequent suppression of circulating CD19+ B-cell to 0%. Re-treatment was then planned after 6 to 9 months. Nine months later (month 13), since CD19+ B-cells levels were 2%, a third 1 g infusion was performed. Ten months later, CD19+ B-cells levels were 3% to 4% and a fourth 1 g infusion was performed (month 23). No side effects occurred in any infusion. No more relapses have occurred for almost 3 years, and neurological examination showed a slow progressive improvement, with stability from month 5 until now: the gait is slightly paraparetic and possible without aid or rest >500 m; muscular strength, tone, and osteo-tendon reflexes are all normal; hypesthesia and dysesthesia with T4 sensory level is bilaterally improved; and the visual acuity is still reduced in both eyes but has improved since the onset of the disease. The clinical stabilization was confirmed by repeated MRI scans of the brain and total SC. During the last MRI of the SC, performed in month 13, we observed no new lesions, no contrast enhancements or T1-hypointensity, dimensional reduction of the left parietal subcortical WM lesion, and thickness reduction of the thoracic SC lesion located posteriorly to the T4-T6 VSs. Moreover, no cortical lesions were detected on a double inversion recovery sequence performed in the last MRI scan.

**Table 1.** Infectious Diseases Diagnostic Assay.

S-VDRL	Negative
L-VDRL	Negative
S-TPHA	Negative
S-anti-*Toxoplasma gondii* IgM/IgG	Negative
S-anti-*Borrelia burgdorferi* IgM/IgG	Negative
L-anti-*Borrelia burgdorferi* IgM/IgG	Negative
L-microbiological examination and bacterial culture	Negative
QuantiFERON test	Negative
L-adenovirus, HHV-6/7/8, HSV-1/2, parvovirus B19, VZV, CMV, enterovirus, JC/BK virus, HIV, HTLV-1, enterovirus, measles virus, West Nile virus (PCR)	Negative
S-anti-measles virus IgM	Negative
S-anti-measles virus IgG	positive (22 UA/mL)
S-anti-HSV 1/2 IgM	Negative
S-anti-HSV 1/2 IgG	positive (26.3 index)
S-anti-VZV IgM	Negative
S-anti-VZV IgG	positive (420 mU/mL)
S-anti-EBV VCA IgM	Negative
S-anti-EBV early antigen IgG	Negative
S-anti-EBV VCA early antigen IgG	positive (34 U/mL)
S-anti-EBV EBNA IgG	positive (516 U/mL)
S-anti-HIV 1/2 IgM/IgG	Negative

Abbreviations: S, serum; VDRL, venereal disease research laboratory test; L, cerebrospinal fluid; TPHA, *Treponema pallidum* Hemagglutination Assay; anti, antibodies; IgM/G, immunoglobulins M/G; HHV, human herpes virus; HSV, herpes simplex virus; VZV, varicella-zoster virus; CMV, cytomegalovirus; JC, John Cunningham virus; HIV, human immunodeficiency virus; HTLV, human T-lymphotropic virus; PCR, polymerase chain reaction; UA, allergenic unit; U, unit; EBV, Epstein-Barr virus; VCA, virus capsid antigen; EBNA, Epstein-Barr nuclear antigen.

**Table 2.** Autoimmune Diseases Diagnostic Assay.

S-ANA	Negative
S-anti-ENA (U1RNP, Sm, SSA/Ro, SSB/La, CENP-B, SCL70, Jo1)	Negative
S-anti-nDNA	Negative
S-ANCA	Negative
S-AMA	Negative
S-ASMA	Negative
S-APCA	Negative
S-ACA IgM/IgG	Negative
S-anti-TPO	Negative
S-anti-TG	Negative
S-ACE	Normal

Abbreviations: S, serum; ANA, antinuclear antibodies; anti, antibodies; ENA, extractable nuclear antigens; U1RNP, U1 ribonucleoprotein; Sm, Smith; SSA, Sjögren’s syndrome–related antigen A; SSB, Sjögren’s syndrome–related antigen B; CENP-B, centromere protein B; SCL70, scleroderma antigen; Jo1, anti-histidyl–tRNA synthetase; nDNA, native DNA; ANCA, antineutrophil cytoplasmic antibodies; AMA, antimitochondrial antibodies; ASMA, anti-smooth muscle antibodies; APCA, anti-gastric parietal cell antibodies; ACA, anticardiolipin antibodies; IgM/G, immunoglobulins M/G; TPO, thyroid peroxidase; TG, thyroglobulin; ACE, angiotensin converting enzyme.

## Discussion and Conclusion

After the first clinical event, represented by an acute bilateral ON and a myelitis, making a diagnosis of NMO was not possible according to the 2006 Wingerchuk diagnostic criteria,^5^ because of the lack of serum AQP4-IgG and longitudinally extensive transverse myelitis (LETM; Table 3). We could not even make a definite diagnosis of NMOSD according to the 2015 criteria for NMOSD AQP4-IgG^neg^ patients,^2^ because the required association between acute myelitis and LETM was not fulfilled, even though, on MRI, both optic nerves presented a T2-hyperintense and T1-Gd+ lesion in their posterior part, involving the optic chiasm (Table 4). Even if possible according to the 2010 McDonald criteria,^6^ a diagnosis of MS was unlikely, because of the absence of most MS-typical clinical and radiological features apart from short myelitis (eg, monolateral and mild-to-moderate ON, with spontaneous or steroid-induced recovery of visual acuity; asymptomatic, extending <1 VS, peripheral WM lesions on SC MRI; cortical, periventricular, or juxtacortical WM lesions on brain MRI; type 2-OBs). MRI lesions pattern could help in the differential diagnosis of CNS demyelinating diseases (especially MS). LETM lesions are the most specific neuroimaging marker of NMOSD and are very uncommon in adult MS patients.^5^ These lesions are usually symptomatic, with an extension ⩾3 VSs, T1-Gd+, and localized in the central SC gray matter (as NMOSD are currently considered astrocytopathies rather than disorders of myelin) and in the upper thoracic SC segments^7^; in contrast, MS SC lesions are usually asymptomatic, with an extension ⩽1 VS, T1-Gd−, and localized in the peripheral WM and in the cervical SC segments.^2,7^ The timing of MRI scan may be very important for the demonstration of LETM: in fact, lesions extending <3 VSs could be detected if MRI is performed too early or too late in the evolution of acute myelitis, or after immunosuppressive treatment, because a LETM lesion may fragment into multiple shorter lesions.^2,8^ Consequently, the MRI scan must be carried out as soon as possible after the onset of the first symptoms and before initiating an immunosuppressive treatment. It is likely that this factor could have limited the detection of LETM in the MRI of our patient. Unilateral or bilateral increased T2-signal or T1-gadolinium enhancement within the optic nerve or optic chiasm, relatively long lesions (ie, extending more than half the distance from orbit to chiasm), together with the lesions involving the posterior part of the optic nerves or the chiasm are associated with NMOSD.^2^ Cortical lesions, detectable by double inversion recovery sequences, are atypical for NMOSD and typical for MS.^2^ Nonspecific brain small lesions (<3 mm) and large confluent WM lesions, with tendency to shrink and even disappear, are common findings in NMOSD (35% to 84%).^2^ Patients with NMOSD are less likely to develop clinically silent MRI lesions than patients with MS.^7^ Moreover, most studies show that nonlesional tissue damage (measured by diffusion tensor MRI), which is rather common in MS, does not occur in NMOSD. This supports the hypothesis that, unlike MS, NMOSD may be a lesion-dependent disease that produces relapses without a progressive clinical phase accompanied by a generalized neurodegeneration.^9^ Combinations of NMOSD and MS features (ie, severe ON and short myelitis) are not uncommon in patients suspected for NMOSD. Therefore, it has been proposed that different degrees of diagnostic certainty could be particularly useful in such phenotypes.^10^ In this perspective, our patient was more likely to have NMOSD than MS, mainly because of the following features: (1) bilateral and severe ON (almost blindness), with little recovery of visual acuity—which occurs neither spontaneously nor after steroid treatment—and association with a T2-hyperintense and T1-Gd+ lesion in both posterior optic nerves, involving the optic chiasm; (2) myelitis associated to a symptomatic lesion on MRI, which was T1-Gd+, localized in the central SC gray matter and in the upper thoracic SC segments; (3) absence of MS-typical brain cortical, periventricular (“Dawson’s fingers”) or juxtacortical WM lesions, and of SC asymptomatic, extending <1 VS, T1-Gd−, peripheral WM lesions on MRI; (4) absence of MS-typical OBs; (5) absence of MS-typical clinical and radiological dissemination in space and time of the disease. Patients suspected for NMOSD who are AQP4-IgG^neg^ could also represent a diagnostic challenge despite the use of the best available assays. AQP4-IgG levels tend to increase during relapses and to decrease when immunosuppressive drugs are administered.^2^ These factors could have limited the detection of AQP4-IgG in the serum of our patient. Dosing AQP4-IgG as closely as possible to the clinical manifestations of a suspected NMOSD, and before initiating an immunosuppressive treatment, could represent a more correct and reliable diagnostic workup. In AQP4-IgG^neg^ patients, the presence of myelin oligodendrocyte glycoprotein-IgG and aquaporin-1 (AQP1)-IgG, which could cause a similar clinical picture, should be ruled out.^1^ In our case, the former has been excluded, while the latter has not been tested. AQP4-IgG^neg^ patients tend to have lower relapse rate and lower disability according to many studies.^11^ Further research needs to be carried out on the possible differences in neuroimaging features between AQP4-IgG^pos^ and AQP4-IgG^neg^ NMOSD. An induction therapy, made by immunosuppressive agents, is currently considered the best approach to treat inflammatory autoimmune diseases with a high risk of relapses-derived disability, such as NMOSD and MS.^10,12^ The main treatment objectives for NMOSD are the improvement of relapse-associated symptoms and the prevention of relapses. Therefore, rituximab is increasingly recommended as a first-line treatment for these diseases,^13^ even if class I studies are still lacking.^3^ Early immunosuppression is fundamental for AQP4-IgG^pos^ and AQP4-IgG^neg^ patients, because AQP4-IgG^neg^ patients risk a conversion to the typical AQP4-IgG^pos^ NMOSD phenotype and, thus, of relapses. To date, a standardized rituximab treatment protocol in NMOSD does not exist, but there is general agreement that an induction therapy with infusion of 1 g of the drug for 2 weeks can provide a deep B-cell depletion and therefore a faster and long-lasting stabilization of the disease.^14^ For the maintenance therapy, a fixed reinfusion of 1 g of the drug every 6 to 9 months is recommended in order to obtain 2 to 4 years of disease stability in 81% to 84% of patients,^3,13,15^ considering that B-cell repletion, and a major relapse risk, takes place after at least 6 months. Monitoring CD19/20+ B-cell count could be a practical way to mark the immunosuppressed state of the patient and the therapeutic efficacy of rituximab. In conclusion, this case report and the related literature suggest that, even in absence of AQP4-IgG and LETM, the concomitant onset of severe bilateral ON and short myelitis (lesions extending <3 VSs), in absence of typical clinical and radiological MS features, should raise a strong suspicion of NMOSD, and that rituximab could be the most effective treatment for these diseases.^4^

**Table 3.** Proposed Diagnostic Criteria for NMO (2006).

Optic neuritis
Acute myelitis
At least 2 of 3 supportive criteria
1. Contiguous spinal cord MRI lesion extending over ⩾3 vertebral segments
2. Brain MRI not meeting diagnostic criteria for multiple sclerosis
3. NMO-IgG seropositive status

Abbreviations: NMO, neuromyelitis optica; MRI, magnetic resonance imaging; IgG, immunoglobulin G.

**Table 4.** NMOSD Diagnostic Criteria for Adult Patients (2015).

Diagnostic criteria for NMOSD with AQP4-IgG
1. At least 1 core clinical characteristic
2. Positive test for AQP4-IgG using best available detection method (cell-based assay strongly recommended)
3. Exclusion of alternative diagnoses
Diagnostic criteria for NMOSD without AQP4-IgG or NMOSD with unknown AQP4-IgG status
1. At least 2 core clinical characteristics occurring as a result of one or more clinical attacks and meeting all of the following requirements:
a. At least 1 core clinical characteristic must be optic neuritis, acute myelitis with LETM, or area postrema syndrome
b. Dissemination in space (2 or more different core clinical characteristics)
c. Fulfillment of additional MRI requirements, as applicable
2. Negative tests for AQP4-IgG using best available detection method, or testing unavailable
3. Exclusion of alternative diagnoses
Core clinical characteristics
1. Optic neuritis
2. Acute myelitis
3. Area postrema syndrome: Episode of otherwise unexplained hiccups or nausea and vomiting
4. Acute brainstem syndrome
5. Symptomatic narcolepsy or acute diencephalic clinical syndrome with NMOSD-typical diencephalic MRI lesions
6. Symptomatic cerebral syndrome with NMOSD-typical brain lesions
Additional MRI requirements for NMOSD without AQP4-IgG and NMOSD with unknown AQP4-IgG status
1. Acute optic neuritis: Requires brain MRI showing (a) normal findings or only nonspecific white matter lesions, or (b) optic nerve MRI with T2-hyperintense lesion or T1-weighted gadolinium enhancing lesion extending over more than one-half the optic nerve length or involving optic chiasm
2. Acute myelitis: Requires associated intramedullary MRI lesion extending over ⩾3 contiguous segments (LETM) or ⩾3 contiguous segments of focal spinal cord atrophy in patients with history compatible with acute myelitis
3. Area postrema syndrome: Requires associated dorsal medulla/area postrema lesions
4. Acute brainstem syndrome: Requires associated periependymal brainstem lesions

Abbreviations: NMOSD, neuromyelitis optica spectrum disorders; AQP4, aquaporin-4; IgG, immunoglobulin G; LETM, longitudinally extensive transverse myelitis lesions; MRI, magnetic resonance imaging.

## References

[1] Jasiak-Zatonska M Kalinowska-Lyszczarz A Michalak S Kozubski W. The immunology of neuromyelitis optica-current knowledge, clinical implications, controversies and future perspectives. Int J Mol Sci. 2016;17:273. doi:10.3390/ijms17030273 PMC4813137 26950113

[2] Wingerchuk DM Banwell B Bennett JL et al; International Panel for NMO Diagnosis. International consensus diagnostic criteria for neuromyelitis optica spectrum disorders. Neurology. 2015;85:177-189. doi:10.1212/WNL.0000000000001729 26092914 PMC4515040

[3] Collongues N de Seze J. An update on the evidence for the efficacy and safety of rituximab in the management of neuromyelitis optica. Ther Adv Neurol Disord. 2016;9:180-188. doi:10.1177/1756285616632653 27134673 PMC4811013

[4] Kim SH Huh SY Lee SJ Joung A Kim HJ. A 5-year follow-up of rituximab treatment in patients with neuromyelitis optica spectrum disorder. JAMA Neurol. 2013;70:1110-1117. doi:10.1001/jamaneurol.2013.3071 23897062

[5] Wingerchuk DM Lennon VA Pittock SJ Lucchinetti CF Weinshenker BG. Revised diagnostic criteria for neuromyelitis optica. Neurology. 2006;66:1485-1489. doi:10.1212/01.wnl.0000216139.44259.74 16717206

[6] Polman CH Reingold SC Banwell B et al Diagnostic criteria for multiple sclerosis: 2010 revisions to the McDonald criteria. Ann Neurol. 2011;69:292-302. doi:10.1002/ana.22366 21387374 PMC3084507

[7] Kim HJ Paul F Lana-Peixoto MA et al; Guthy-Jackson Charitable Foundation NMO International Clinical Consortium Biorepository. MRI characteristics of neuromyelitis optica spectrum disorder: an international update. Neurology. 2015;84:1165-1173. doi:10.1212/WNL.0000000000001367 25695963 PMC4371410

[8] Asgari N Skejoe HPB Lillevang ST Steenstrup T Stenager E Kyvik KO. Modifications of longitudinally extensive transverse myelitis and brainstem lesions in the course of neuromyelitis optica (NMO): a population-based, descriptive study. BMC Neurol. 2013;13:33. doi:10.1186/1471-2377-13-33 23566260 PMC3622587

[9] Pichiecchio A Tavazzi E Poloni G et al Advanced magnetic resonance imaging of neuromyelitis optica: a multiparametric approach. Mult Scler. 2012;18:817-824. doi:10.1177/1352458511431072 22183930

[10] Juryńczyk M Weinshenker B Akman-Demir G et al Status of diagnostic approaches to AQP4-IgG seronegative NMO and NMO/MS overlap syndromes. J Neurol. 2016;263:140-149. doi:10.1007/s00415-015-7952-8 26530512 PMC4816597

[11] Jarius S Ruprecht K Wildemann B et al Contrasting disease patterns in seropositive and seronegative neuromyelitis optica: a multicentre study of 175 patients. J Neuroinflammation. 2012;9:14. doi:10.1186/1742-2094-9-14 PMC3283476 22260418

[12] Rommer PS Dörner T Freivogel K et al; GRAID Investigators. Safety and clinical outcomes of rituximab treatment in patients with multiple sclerosis and neuromyelitis optica: experience from a national online registry (GRAID). J Neuroimmune Pharmacol. 2016;11:1-8. doi:10.1007/s11481-015-9646-5 26589235

[13] Zéphir H Bernard-Valnet R Lebrun C et al Rituximab as first-line therapy in neuromyelitis optica: efficiency and tolerability. J Neurol. 2015;262:2329-2335. doi:10.1007/s00415-015-7852-y 26194198

[14] Annovazzi P Capobianco M Moiola L et al Rituximab in the treatment of neuromyelitis optica: a multicentre Italian observational study. J Neurol. 2016;263:1727-1735. doi:10.1007/s00415-016-8188-y 27286847

[15] Radaelli M Moiola L Sangalli F et al Neuromyelitis optica spectrum disorders: long-term safety and efficacy of rituximab in Caucasian patients. Mult Scler. 2016;22:511-519. doi:10.1177/1352458515594042 26199350

